# Implementation cost analysis of collaborative care for perinatal mental health in community health centers

**DOI:** 10.21203/rs.3.rs-5256122/v1

**Published:** 2024-11-27

**Authors:** Tess Grover, Ian M Bennett, Mark Campbell, Melinda Vredevoogd, Lisa Saldana

**Affiliations:** University of Washington Seattle Campus: University of Washington; University of Washington Seattle Campus: University of Washington; Chestnut Health Systems Inc; University of Washington Seattle Campus: University of Washington; Chestnut Health Systems Inc

**Keywords:** perinatal depression, collaborative care, implementation costs, integrated behavioral health

## Abstract

**Background:**

Although costs are of key importance to clinic leadership when considering adoption of new programs, few studies examine real-world resource needs associated with implementing complex interventions for chronic conditions in primary care. This analysis sought to identify the costs necessary to implement the evidence-based collaborative care model (CoCM), an integrated behavioral health program for common mental disorders in primary care.

**Methods:**

Ten federally qualified health centers (FQHCs) adopted CoCM as part of a larger national randomized trial evaluating implementation strategies for CoCM when adapted for perinatal mental health. The Cost of Implementing New Strategies (COINS) tool was used to assess implementation costs associated with activities completed by sites as they progressed through the implementation process. National wage norms were used to calculate cost estimates for staff time.

**Results:**

On average, clinics spent $40,778 (SD=$30,611) on implementation, with clinics ranging widely from $4,502 to $103,156. Three out of 10 participating clinics achieved competency in the intervention during the 2-year implementation period. Costs among competent clinics ranged from $20,944 to $65,415 (mean=$41,788). Clinics that did not achieve competency were more varied, with both the lowest and highest resource use. Significant staff effort was required to complete all implementation stages; clinical staff and program champions showed greatest effort.

**Conclusions:**

Site implementation costs for this complex behavioral health intervention were substantial and varied dramatically, particularly among sites who did not achieve competence. Additional work is needed to identify optimal site resource investment related to implementation success for CoCM.

**Trial registration:**

ClinicalTrials.gov.NCT02976025. Registered on November 23, 2016.

## Background

Perinatal mood and anxiety disorders (PMAD) are common, undertreated, and affect both parents and their infants during pregnancy and the first year after childbirth [1-3]. People who are pregnant or postpartum are at risk for major depressive disorder or dysthymia, with up to 16% experiencing symptoms of depression during pregnancy [4] and up to 14% experiencing symptoms of anxiety [5]. Perinatal depression is more than twice as prevalent in patients who are low-income than other patients [6]. Suicide, most often among patients with depression, is a leading cause of maternal mortality [7, 8], and mental health conditions are linked to 11% of pregnancy-related deaths [9]. Despite these significant deleterious outcomes, patients from low income or racial and/or ethnic minority backgrounds are less likely to be identified and treated for PMAD than other patients [10, 11]. Systematic efforts to address the mental health needs of parents during the perinatal period in general, and within these high-risk populations specifically, are not widely implemented [10, 11].

Federally qualified health centers (FQHCs), are federally supported safety net sites serving disproportionately high numbers of low income and race/ethnic minority populations that have historically been underserved. These sites provide multi-disciplinary maternal-child and primary care allowing for opportunities to address PMAD through continuity across the pre/inter-pregnancy, prenatal, and postpartum transitions [12]. Yet, few evidence-based interventions focused on perinatal mental health are employed within primary care settings. The collaborative care model (CoCM) is an evidence-based, behavioral health integration intervention to support the treatment of common behavioral health conditions in primary care settings [13]. CoCM leverages existing behavioral health professionals working in primary care clinics and additional specialty mental health consultants (typically psychiatrists or psychiatric nurse practitioners) to support the patient through team-based care [13-15]. CoCM has been tested in more than 80 randomized controlled trials in the US and internationally, and meta-analyses of these studies indicate that it consistently improves on care as usual, including in perinatal populations [16-22]. Because of the high level of evidence, the Center for Medicare and Medicaid Services (CMS) has promulgated billing codes to support activities related to CoCM [23-25]. Despite these reimbursement opportunities and demonstrated improved outcomes in patients with perinatal depression, CoCM is not yet widely implemented within clinics that serve this population [20].

One barrier to the implementation of new interventions is the perceived resource needs associated with implementation, including training, change management, and salary costs for time spent on this work [26]. This is particularly true for complex interventions such as CoCM that involve coordination across multiple providers, managers, and diverse support staff.[27] Previous studies examining cost associated with CoCM primarily examined cost effectiveness of the intervention, finding improved clinical outcomes at no greater cost than usual care [28, 29]. Cost-benefit analyses have shown that every dollar spent on CoCM for geriatric depression resulted in $6 long-term healthcare costs saved per patient [30]. Other studies have found that CoCM produces lower annualized total healthcare costs per patient than usual care [31] and less outpatient health services costs than usual care [32]. While helpful for public policy financing decisions, these analyses do not address the implementation cost for integrating the intervention into clinics, which is critical to individual site choices regarding the adoption of clinical innovations. Despite the positive cost outcomes of CoCM in rigorous trials for treating depression, the unknown real-world implementation costs–including the costs of building the infrastructure and environment needed to successfully deliver CoCM–pose a barrier to adoption. By understanding the implementation costs of CoCM for perinatal depression, appropriate planning and support can be provided for clinics serving patients at risk for PMAD. In the current study, we wished to capture the real-world costs associated with implementing CoCM for perinatal depression in health centers serving patients in pregnancy and the year postpartum from historically underserved populations and at high risk of PMAD. Those costs include staffing based on the amount of time various clinic team members spent on implementation activities, practice facilitation, coaching, and training.

## Methods

### Participants:

The Maternal Infant Dyad Implementation (MInD-I) study was designed to evaluate strategies for helping sites implement CoCM for perinatal depression and anxiety (NCT02976025: PI Bennett). The study was reviewed and approved by the University of Washington Institutional Review Board (IRB). Sites were recruited from the OCHIN national primary care informatics network comprised primarily of Federally Qualified Health Centers (FQHCs) and FQHC look-alikes [12]. OCHIN sites are part of a national collaborative of over 500 primary care clinics in 47 states that share an iteration of the Epic© electronic health record. Clinics were enrolled in the study based on two criteria: 1) use of the shared Epic© electronic medical record for perinatal care and 2) a minimum of 50 perinatal patients served annually.

Ten FQHCs agreed to participate in the first cohort of the project from April 2016 to April 2020, including 4 sites in California, 2 sites in Massachusetts, 1 site in Indiana, 1 in Texas, 1 in Wisconsin, and 1 in Oregon. Sites ranged in size, serving 59–1,245 unique perinatal patients annually, with a mean of 406 perinatal patients served per year. On average, 72% of patients served by the sites identified as racial and/or ethnic minority patients (as reported in annual health center data) and 89% were at or below 200% of the federal poverty guideline (Table 1).

### Implementation Strategies

Participating clinics received implementation support and registry training from experts in practice facilitation based at OCHIN and clinical training and expert consultation from CoCM trainers and consultants employed by the Advancing Integrated Mental Health Solutions (AIMS) Center at the University of Washington. Practice facilitation began roughly 3 months prior to launching care and was intended to orient sites to CoCM, help identify staff for collaborative care team roles, and develop team readiness and clinical workflows. Core team members, including care managers, psychiatric consultants, PCP (primary care provider) champions, project leads, and the Epic© site specialist from each clinic attended a 2-day in-person CoCM training prior to program launch. The core care team received 1 hour of practice facilitation support monthly. Care managers met once a month for training webinars, and psychiatric consultants met quarterly for calls with an expert CoCM psychiatrist trainer. Clinics were provided with online training resources via an online learning management system (LMS), but use of these resources varied among clinics and roles. Clinics were permitted to configure the CoCM team for their clinical context, as long as key components were maintained, resulting in variation in the types and quantities of staff participating in the implementation of MInD-I at each site. Four of the sites received additional support over the first 12 months after .launching services in the form of Longitudinal Remote Coaching (LRC) by an experienced psychiatric consultant who observed the work of the local teams during select systematic case reviews and provided feedback [33].

### Measures

The Cost of Implementing New Strategies (COINS) approach is a method for mapping resources needed for implementation efforts of sites (clinics/agencies/systems) adopting new interventions or practices [34, 35]. Implementation costs are mapped onto activities operationalized within the Stages of Implementation Completion (SIC©) [36, 37]. The SIC is a measure of implementation process that defines, captures, and tracks necessary implementation activities by newly adopting sites across eight stages, from Engagement (Stage 1) to Competency (Stage 8). Each of these stages falls within three well-established phases of implementation: Pre-implementation, Implementation, and Sustainment [38].

The SIC has been rigorously evaluated and developed for use for the implementation of evidence-based practices (EBPs) [37, 39, 40]. Although the 8 implementation stages are standard across practices, activities within the stages are tailored or customized to the practice being monitored. The SIC previously has been adapted for CoCM [36], which in turn was customized further for the MInD-I implementation strategies that were integrated within CoCM. During the course of the MInD-I implementation, sites incurred resources and costs as they progressed through the implementation process, including assessment of feasibility (SIC Stage 2), readiness planning (SIC Stage 3), hiring and training of staff (SIC Stage 4), delivering the program with fidelity (SIC Stages 5–7), and ultimately achieving competency to sustain the program (SIC Stage 8). The COINS tool provides a validated approach to collect the costs and resources involved in completing the implementation strategies associated with each of these stages.

The COINS tool is web-based, allowing for easier real-time data entry and management. To prepare for programming of the MInD-I COINS, implementation activities were identified on the MInD-I SIC where resource use might occur. The type of positions and/or roles that might be involved in completing each activity were defined and programmed to capture which roles were involved for each site. The COINS system prompted the data collector to capture human resources (i.e., amount of time and position), and fixed expenditures (e.g., rent, computers, advertising) that were required to implement MInD-I. The majority of the costs in the Pre-Implementation Phase resulted from labor resources on the unique activities, with none of the sites incurring associated fixed, infrastructure or assigned FTE (full-time equivalent) costs. Implementation Phase costs primarily resulted from clinical staff FTE assigned to the implementation, in addition to the direct resources spent on each activity at the site.

### Data Collection

Data were collected by a member of the research team (the first author) from each of the 10 participating sites during the site support period from April 2016 to April 2020. COINS data were collected through remote conversations among the data collector, implementation facilitators, and the sites. Additionally, site and purveyor organization travel costs were collected via project invoices. Data regarding the number of patients served by the MInD-I program came from ADVANCE (Accelerating Data Value Across a National Community Health Center Network), a PCORnet clinical trials network that includes data from community health centers.

## Results

### Site Costs:

The COINS method described above produces several different results that can be compiled to generate the total cost of implementation: 1) fixed expenses associated with the implementation, 2) staff hours associated with the discrete implementation activities described in the SIC, and 3) MInD-I FTE allocation during the implementation process. Fixed expenses in this context refers to rent/office space, computers, office supplies, and other non-employee based expenses incurred to support the intervention. Because MInD-I was incorporated into existing clinic settings for the project, no additional fixed expenses were reported. Thus, all resource costs to implement MInD-I within the participating clinics were strictly incurred by staff hours.

Table 2 shows the start and end dates for the MInD-I implementation at the 10 different sites, the duration of time spent from the beginning of the implementation to completion (i.e., achievement of competency or discontinuation) of the site, the highest SIC stage the site reached during implementation, and whether or not the site was deemed competent in the MInD-I intervention. To address study timeline limitations, sites were given a fixed contract time period of 2 years to complete the implementation. As shown, three sites achieved “Competent” delivery of MInD-I within that timeframe.

Table 3 shows the provider and staff roles at each clinic that were identified as having participated in implementation activities operationalized on the MInD-I SIC. As shown, the average hours spent on MInD-I implementation activities varied by position type across the implementation stages. The majority of leadership and staff hours spent toward the implementation were during Stage 3 (Readiness) through Stage 7 (Ongoing Service Delivery and Fidelity Monitoring), including activities related to setting up infrastructure, completing fidelity monitoring protocols, and establishing MInD-I within the clinic. All 10 sites endorsed using clinical staff, a program champion, and support staff, with the majority of implementation activity hours being completed by these three positions. The COINS also assesses the FTE assignment of each position. The Bureau of Labor Statistics (BLS) national median hourly wage information was mapped onto each position to obtain a quantifiable estimate of the resources involved. The BLS wages allowed for an average national estimate for the different positions, reducing the potential to over- or underestimate between-site costs due to wage discrepancies between site locations’ local wages.

Table 4 quantifies the implementation activity costs for MInD-I throughout the implementation process by combining reported hours and BLS wage data for identified positions involved in implementation. Minimal staffing resources were identified in Stages 1 (Engagement) and 2 (Feasibility Assessment), with resource use increasing in Stage 3 (Readiness). The cumulative total prior to Stage 4 represents costs incurred during the Pre-Implementation Phase (Stages 1–3). Costs ranged from $1,594 – $7,810 across sites for this phase. Costs for the sites began to diverge more during the Implementation Phase (Stages 4–7). As depicted in Table 2, this is due to variation in the duration of the implementation among sites. Site A, for example, achieved competency in 14.7 months at an observed cost of $20,944, whereas Site C spent 23.7 months (without reaching competency) at a cost of $73,580.

The total cost to implement MInD-I was, on average, $40,778 (SD=$30,611) per site with a wide range from $4,502 – $103,156 across sites.

### Implementation Training and Facilitation Costs

Costs associated with expert consultation and training provided to the sites are shown in Table 5. The total charges for training and practice facilitation were $444,251, resulting in a cost of $44,425 for each site across the cohort. Combining the average staffing cost of $40,778 per MInD-I clinic with the $44,425 paid by the grant for travel, training, and expert consultation, the total cost per site would have been $85,203 if the grant had not covered the aforementioned training components.

## Discussion

Implementation costs can be difficult to capture but if left undefined can lead to uninformed decisions regarding whether or not to implement a new program and where to direct resources. Evidence from the first 10 clinics recruited to implement the evidence-based CoCM intervention for team-based integrated perinatal mental health care suggested that successful programs were relatively efficient (utilizing neither too many nor too little resources) in their implementation approach. Site implementation costs for this complex behavioral health intervention were substantial and varied dramatically, particularly among sites that did not achieve competence— those sites incurred costs that were either considerably higher or lower than sites that did achieve competence, indicating inefficient use of resources or lack of dedication to the implementation. Of note, one site (site J) discontinued relatively quickly (10.9 months), spending less ($19,464) than the overall average implementation costs, reflecting the relative savings associated with the decision to end implementation rather than continue with little likelihood of success. Further, our study demonstrated that the COINS method can successfully assess cost and resource variations across clinics implementing a complex, multicomponent implementation strategy and that successful and cost viable implementation of CoCM is possible within 2 years.

One limitation of this study is that it did not directly track ways in which sites could have recouped implementation costs. Currently, 16 states allow for Medicaid billing for the select Current Procedural Terminology (CPT) codes associated with CoCM [41]. Billing for CoCM also requires complex tracking procedures novel to most sites such as tracking the number of minutes spent on care coordination over the course of a month, with a requirement that the first month must have at least 36 minutes of billable activity and subsequent months must have at least 31 minutes in order to bill for services. Although it would have been possible for a site participating in MInD-I to recoup the implementation costs of CoCM within the two-year project window, it is more likely that it would take longer. Further research is needed to determine real-world ability of sites to recoup implementation costs through billing practices for newly implemented CoCM services.

Additionally, we recommend more research to better pinpoint which site resource investments contribute most to the successful implementation of CoCM. Knowing more about where sites should invest their time and effort would allow CoCM practice facilitators to strategically coach sites on how best to utilize their resources to more efficiently implement with a higher rate of competence. Cost information can thereby support efforts to expand evidence-based models of care to more patients in need with less financial burden on sites.

## Conclusions

Site leadership must consider implementation costs for complex behavioral health interventions such as CoCM to plan appropriately for implementing these interventions. Sites that failed to successfully implement CoCM for the perinatal population within a 2-year period tended to utilize resources inefficiently, incurring either much higher or much lower costs than sites that achieved competence in this model of care. Further understanding of optimal site resource investment is needed to help support sites seeking to improve behavioral health services through evidence-based practice change.

## Figures and Tables

**Table 1:** MInD-I site baseline characteristics^a^

Site	State	Unique patients per year	Unique perinatal patients per year	Racial and/or ethnic minority patients	Patients at or below 200% of federal poverty level
A	CA	12,144	188	70%	96%
B	CA	11,400	323	95%	99%
C	CA	15,145	469	84%	80%
D	TX	12,032	1345	97%	98%
E	CA	55,159	637	19%	76%
F	WI	11,408	256	93%	96%
G	OR	3359	59	3%	63%
H	IN	10,000	430	90%	98%
I	MA	13,271	180	78%	87%
J	MA	12,410	175	95%	100%
Mean	---	15,633	406	72%	89%

a Source: US Health Resources and Services Administration Data Warehouse (2016 data)

**Table 2:** MInD-I implementing site characteristics

Site	Duration (months)	Highest SIC© stage	Competent	Number of CoCM patients
A	14.7	8	Yes	45
B	25.5	8	No	14
C	23.7	8	No	43
D	20.6	8	No	27
E	33.1	8	Yes	172
F	26.5	8	No	72
G	17.5	8	Yes	15
H	20.3	8	No	21
I	19.6	7	No	36
J	10.9	6	No	0

**Table 3:** Mean hours spent on implementation tasks by site role^a^

Role type	Count	Stage 1	Stage 2	Stage 3	Stage 4	Stage 5	Stage 6	Stage 7	Stage 8	Total hours
		(N = 10)	(N = 10)	(N = 10)	(N = 10)	(N = 10)	(N = 10)	(N = 6)	(N = 2)	
Admin support^b^	1	0	0	2	0	0	0	0	0	2
CEO/director	6	1	1	2	0	0	0	0	0	4
Clinical staff	10	1	1	19	39	25	3	25	6	119
Executives	8	1	0	2	2	0	1	6	0	12
Finance	4	0	0	4	1	0	0	1	0	6
Financial officer	3	0	0	4	0	0	0	0	0	4
Middle management	5	2	1	5	3	0	0	1	0	12
Program champion	10	2	1	21	3	9	1	12	1	50
Support staff	10	0	1	5	0	20	1	5	0	32
Total stage hours	---	7	5	64	48	54	6	50	7	241

a The table represents the average (mean) resources reported per position per site. Parentheses indicate the number of sites reporting resource use for a listed position, or the number of sites represented per stage.

b Includes clerical staff, administrative assistants, and public relations staff.

**Table 4:** Total implementation cost by stage

Site	Activity Total	FTE Total	Cumulative Total
A	$5,557	$15,387	$20,944
B	$3,389	$1,114	$4,502
C	$4,618	$68,962	$73,580
D	$3,903	$16,674	$20,577
E	$4,237	$34,769	$39,006
F	$2,820	$100,336	$103,156
G	$7,625	$57,790	$65,415
H	$7,902	$30,418	$38,320
I	$4,571	$19,622	$24,089
J	$3,447	$16,017	$19,464
Site average	$4,807	$36,109	$40,778

**Table 5:** OCHIN & AIMS consultation and training costs

Type of cost	2017	2018	2019	2020	Total cost
OCHIN & AIMS FTE	$113,446	$133,326	$44,441	$2,027	$293,241
Travel	$9,250	$23,092	$386	---	$32,728
Other (catering, supplies, etc.)	$389	$1,193	$117	---	$1,699
PST training	---	---	$1,900	---	$1,900
Overhead (35%)	$43,080	$55,164	$15,731	$710	$114,684
Total cost	$166,165	$212,774	$62,575	$2,737	$444,252

## Abbreviations

FQHCs: federally qualified health centers
CoCM: collaborative care model
COINS: Cost of Implementing New Strategies
PMAD: perinatal mood and anxiety disorders
CMS: Center for Medicare and Medicaid Services
MInD-I: Maternal Infant Dyad Implementation
PCP: primary care provider
SIC: Stages of Implementation Completion
EBPs: evidence-based practices
FTE: full-time equivalent
ADVANCE: Accelerating Data Value Across a National Community Health Center Network
PCORnet: National Patient-Centered Clinical Research Network
CPT: Current Procedural Terminology
